# Computational screening of nanoparticles coupling to *Aβ*40 peptides and fibrils

**DOI:** 10.1038/s41598-019-52594-8

**Published:** 2019-11-28

**Authors:** Soumyo Sen, Lela Vuković, Petr Král

**Affiliations:** 1 University of Illinois at Urbana-Champaign, NIH Center for Macromolecular Modeling and Bioinformatics, Beckman Institute, Urbana-Champaign, 61801 United States; 2 University of Texas at El Paso, Department of Chemistry and Biochemistry, El Paso, 79968 United States; 3 University of Illinois at Chicago, Departments of Chemistry, Physics, Biopharmaceutical Sciences and Chemical Engineering, Chicago, 60607 United States

**Keywords:** Atomistic models, Nanomedicine, Molecular dynamics

## Abstract

Blocking the formation, growth, and breaking of amyloid fibrils by synthetic nanosystems could provide a treatment of neurodegenerative diseases. With this in mind, here atomistic molecular dynamics simulations are used to screen for nanoparticles (NPs), covered with different mixtures of ligands, including positively and negatively charged ligands, *Aβ*40-cut-peptide, and synthetic inhibitor ligands, in their selective coupling to *Aβ*40 peptides and their fibrils. The simulations reveal that only *Aβ*40-cut-peptide-covered NPs have strong and selective coupling to *Aβ*40 monomers. On the other hand, positive, positive-neutral, Janus, and peptide NPs couple to the beta sheet surfaces of *Aβ*40 fibrils and only the negative-neutral NPs couple to the fibril tips.

## Introduction

In recent years, NPs were used for bio-imaging, diagnostics, and delivery of drugs in biological systems^1–5^. NPs can be designed to selectively interact with and affect the functions of lipids^6,7^, proteins^8,9^, and various bio-molecular complexes. For example, NPs can have strong multivalent and selective interactions with viruses allowing them to act as broad spectrum virucidal drugs^10^.

In principle, NPs could be designed to specifically interact with amyloid fibrils, which are responsible for the development of Alzheimer, Parkinson and other neurological disorders. Some NPs were already shown to induce^11–13^ or prevent^14,15^ fibrillation of amyloid peptides, depending on their sizes, shapes, hydrophobicity and other parameters. It might be particularly interesting to prepare NPs that can affect the formation, growth, and breaking of amyloid fibrils structures^16^, in analogy to polymer-peptide conjugates that can break amyloid fibrils^17^.

Recently, molecular dynamics (MD) simulations were used to examine how selected NPs interact with amyloid peptides and affect their fibrillation by influencing the conformational change and the local concentration of free peptides^12,18–22^. Still, there is a lack of information how the NPs with different surface properties interact with free *Aβ*40 peptides and specifically with a fibril. In this work, we perform systematic atomistic MD simulations to screen NPs of different types selectively coupling with free *Aβ*40 peptides and their fibrils.

## Results and Discussion

**Figure 1:**
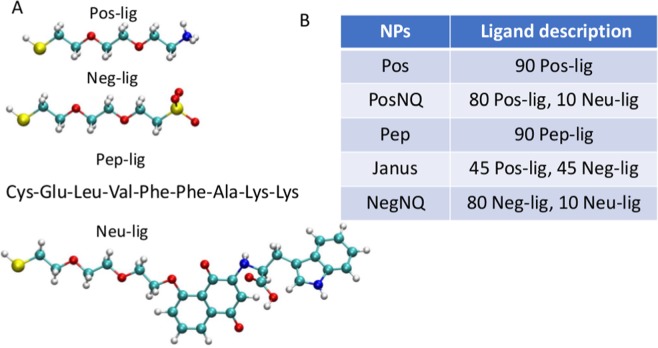
Nanoparticle ligands screened in simulations. (**A**) (Pos-lig) PEG chain terminated with ${-\mathrm{NH}}_{3}^{+}$ group, (Neg-lig) PEG chain terminated with ${-\mathrm{SO}}_{3}^{-}$ group, (Pep-lig) peptide ligand, (Neu-lig) PEG chain terminated with NQTrp (quinone-tryptophan hybrid). Images of ligands created by VMD 1.9.3 software (https://www.ks.uiuc.edu/Research/vmd/). (**B**) The ligand composition in each nanoparticle.

Figure 1(A) shows four ligands which are used to cover NPs in the present modeling: positive (Pos-lig; ${\text{NH}}_{3}^{+}$ terminal group), negative (Neg-lig; ${\text{SO}}_{3}^{-}$ terminal group), neutral (Neu-lig; NQTrp terminal group) and peptide ligands (Pep-lig). Except Pep-lig, all the ligands have two ethylene glycol groups (PEG) to reduce H-bonding between PEG oxygens and ${\text{NH}}_{3}^{+}$ terminal groups (Pos-lig)^6^. NQTrp is a quinone-tryptophan hybrid molecule, which is actually an inhibitor of an *Aβ* fibrillation process^23^. The amino acid sequence in Pep-lig, Cys-Glu-Leu-Val-Phe-Phe-Ala-Lys-Lys, follows the sequence, Lys-Leu-Val-Phe-Phe-Ala-Glu-Asp, present in the exposed region of a *β* sheet of the *Aβ*40 fibril. The charged amino acids (Lys, Glu and Asp) of Lys-Leu-Val-Phe-Phe-Ala-Glu-Asp are replaced with the oppositely charged amino acids (Glu, Lys, Lys) in Pep-lig, while keeping the remaining amino acids identical. The ligands are attached by thiol groups to the Au NP core (from Cysteine in Pep-lig).

Figure 1(B) describes five NPs prepared and simulated, each with a 2.2 nm diameter Au core and 90 attached and homogeneously (except Janus) distributed ligands. Since, *Aβ*40 is overall negative, three NPs are chosen to be positive (Pos, PosNQ and Pep), one is neutral (Janus) and one is negative (NegNQ). PosNQ and NegNQ NPs have 80 Pos-ligs and Neg-ligs, respectively, and 10 Neu-ligs, decreasing the NPs solubility in water. The number of Neu-lig ligands are optimized in such a way that they cannot be clustered due to van der Waals (vdW) coupling between their aromatic rings, since clustering of NQTrp prevents their coupling with the peptides^5^.

**Figure 2:**
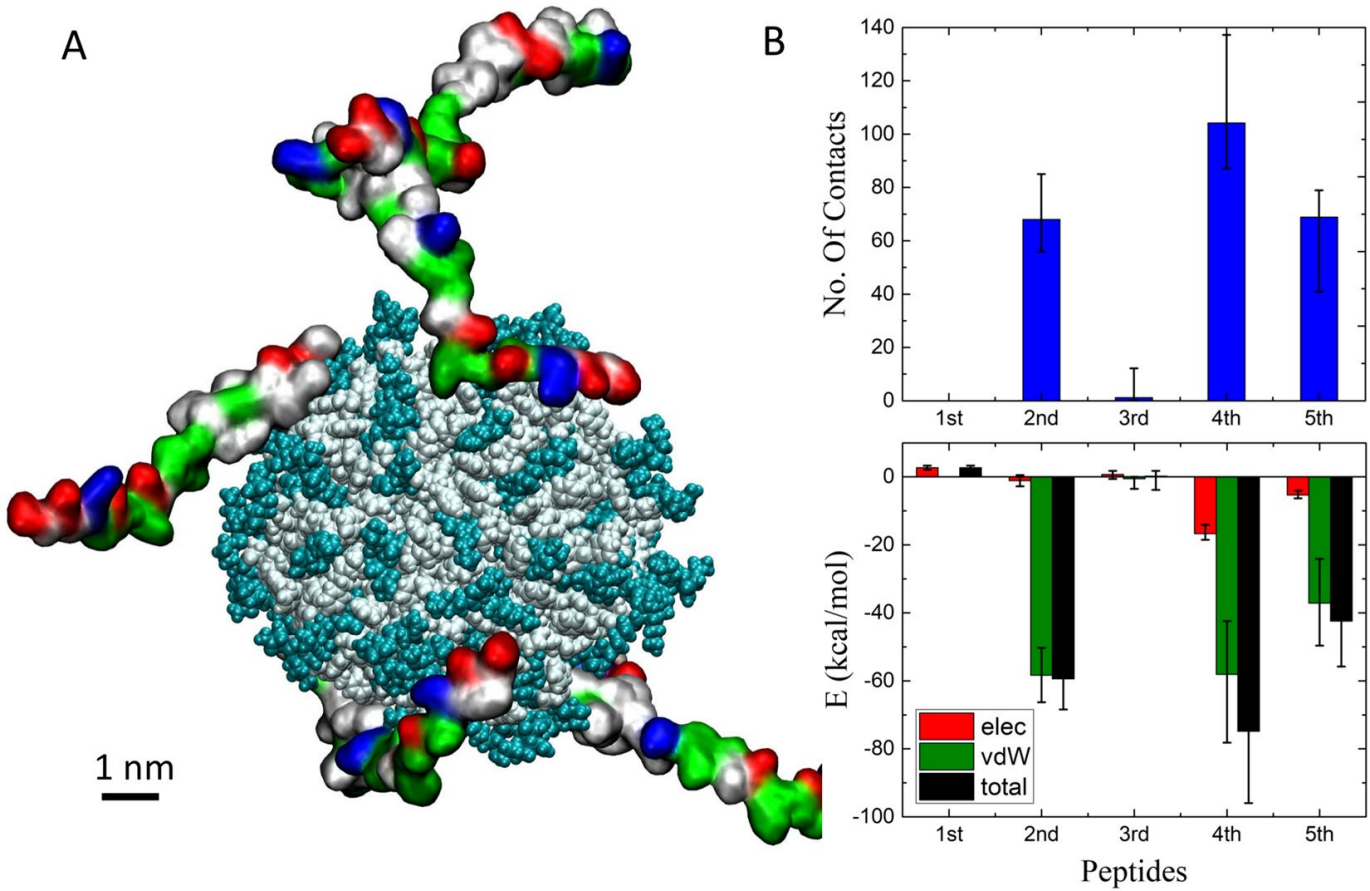
(**A**) A simulation snapshot of Pep NP with five A*β*40 peptides coupled to it, after 100 ns of equilibration. Positive, negative, polar and nonpolar amino acids of free peptides are shown by blue, red, green and white surface, respectively. In the case of Pep NP, terminal positively charged and hydrophobic amino acids are cyan and white, respectively. Images of molecular structures created by VMD 1.9.3 software (https://www.ks.uiuc.edu/Research/vmd/). (**B**) (top) Number of heavy atoms of the A*β*40 peptides within 5 Å of the Pep NP ligands. (bottom) Binding energy of each bound A*β*40 peptide with the Pep NP surface. The numbers in panel B are averaged over the last 5 ns of trajectories.

First, we simulated individual NPs together with (5) freely solvated *Aβ*40 peptides in a 150 mM NaCl solution box of (150 × 150 × 130 Å^3^) dimensions. Most NPs interacted rather weakly with the free peptides. However, after 100 ns of simulations, multiple peptides were found adsorbed on Pep NP (Fig. 2), matching well the peptides structure. To examine the NPs-peptides coupling, we calculated the number of heavy atoms of each peptide within 5 Å of different NPs and their interaction energies with these NPs. The highest number of contacts, 70–110, were observed among the five peptides coupled with Pep NP, individually presented in Fig. 2B (top). These peptides also had the largest coupling energies of −(40–80) kcal/mol (Fig. 2B (bottom)), mostly due to vdW coupling. These results are in line with the observation of peptide polymers capturing *Aβ*42 peptides^24^. The free *Aβ*40 peptides interacted with Pos and PosNQ NPs mainly by Coulombic coupling, while they interacted very little with Janus and NegNQ NPs (Figs. S1 and 2).

**Figure 3:**
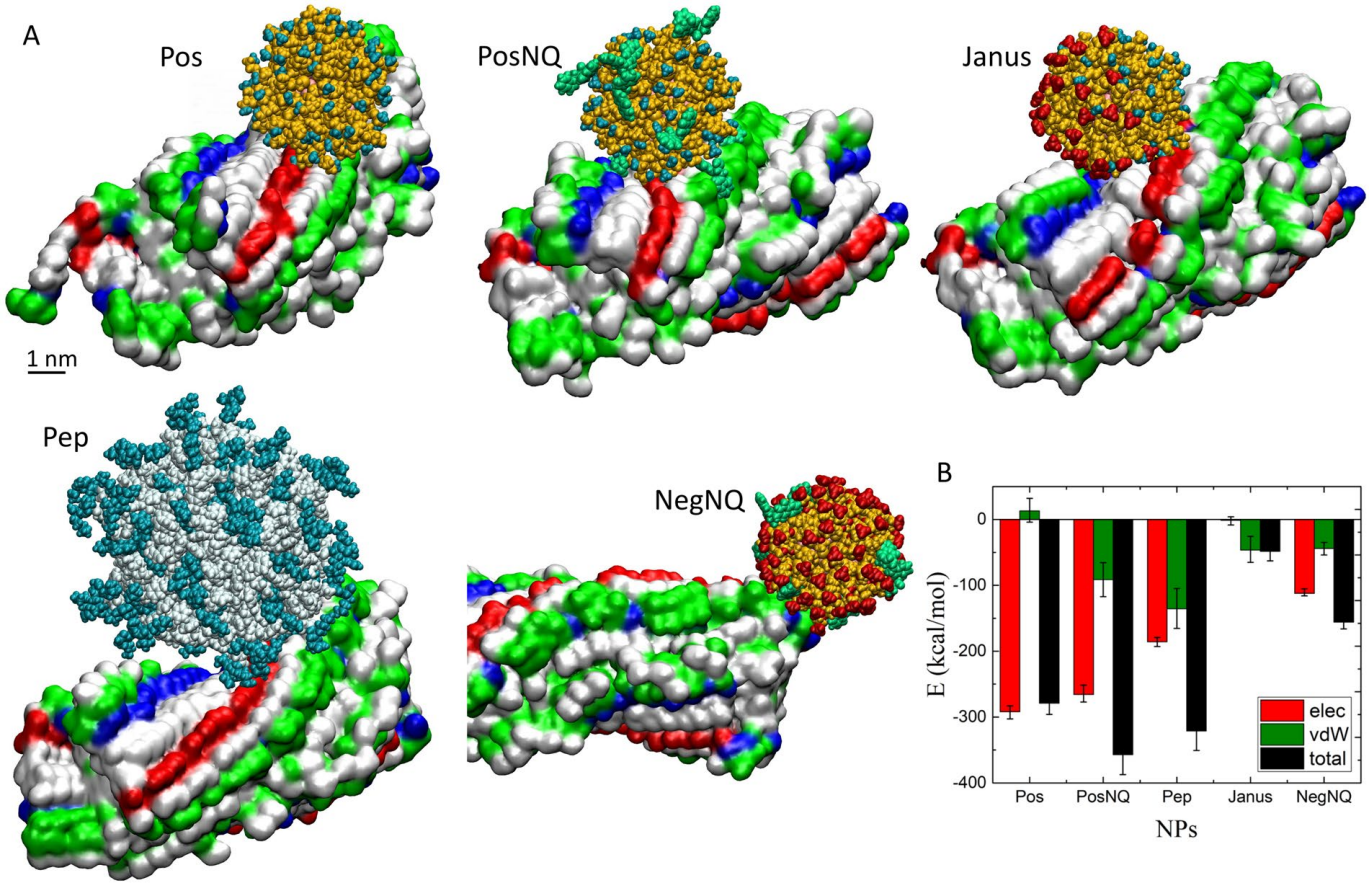
(**A**) NPs adsorbed on a A*β*40 fibril after 100 ns of simulations. Random chains regions of the fibril are not shown. In the case of fibril, positive, negative, polar and nonpolar amino acids are shown by blue, red green and white surface, respectively. For Pos, PosNQ, Janus and NegNQ, PEG chain, ${\text{NH}}_{3}^{+}$, ${\text{SO}}_{3}^{-}$ and NQTrp are shown by yellow, blue, red and green vdW representation, respectively. For Pep NP, terminal positively charged and hydrophobic amino acids are cyan and white, respectively. Pos, PosNQ, Pep and Janus NPs are coupled to the *β* sheet surface and NegNQ NP is coupled at the tip of the fibril. Images of molecular structures created by VMD 1.9.3 software (https://www.ks.uiuc.edu/Research/vmd/). (**B**) Coupling energies of NPs to the specified regions of the A*β*40 fibril, averaged over the last 5 ns of trajectories.

Next, we simulated the coupling of individual NPs to different regions of the *Aβ*40 fibril, consisting of two coupled protofilaments of a 13–14 nm length (29 peptides in each protofilament). Initially, each NP was placed close to one of the two exposed *β* sheet regions within the fibril. At the beginning of the simulation, the NPs were slowly diffusing on the surface, after 90–95 ns of simulations, the positively charged NPs (Pos, PosNQ, Pep) were strongly interacting with the *β* sheet surface, as shown in Fig. 3(A). For example, Pos NP was mostly nested on the negatively charged Glu22, Asp23 and neutral Val24 in the *β* sheet region, and often interacted with Asp1, Ala2 and Glu3 in the random chain regions of the fibril. All these interactions between Pos NP and the fibril gave a strong Coulombic coupling energy (on average of −290 kcal/mol), but a very small vdW decoupling energy (on average of 13 kcal/mol), as displayed in Fig. 3(B) for all NPs.

A rather different situation occured in PosNQ NP, having positively charged ammonium and neutral NQTrp terminal groups, differently interacting with the fibril (Fig. 3(A)). The Coulombic coupling energy (on average of −266 kcal/mol) was slightly smaller than in Pos NP, due to a smaller number of positively charged groups, but the vdW coupling energy, mostly due to NQTrp groups, was rather large (on average of −90 kcal/mol). Out of 10 NQTrp groups, 4 were close to the *β* sheet region and interacted with His14, Gln15, Lys16, Leu17, Val18, Phe20, Val24, Gly25, Ser26 and Asn27. Random chains of the A*β*40 fibril were also wrapping PosNQ NP by Asp1, Glu3, Arg5, Asp7, Ser8, Glu9, Tyr10, and Glu11.

Pep NP is more complex and directly designed to match the peptides, but this specific coupling is mostly active for free peptides (Fig. 2). It has an overall positive charge, which helped it to also nest on Glu22 of the fibril (Fig. 3(A)). The Coulombic (on average of −185 kcal/mol) and vdW (on average of −135 kcal/mol) coupling energies between Pep NP and the fibril were about the same (Fig. 3(B)), reflecting the matching structures of both coupled components. Random chains of the A*β*40 fibril were also relatively strongly interacting with the larger Pep NP by Asp1, Glu3, Phe4, Arg5, His6, Asp7, Ser8, Gly9, Tyr10, and Glu11.

Janus NP has asymmetrically distributed charges on its overall neutral surface. Therefore, its positive side mainly interacted with Phe20, Glu22, Asp23 and Val24 in the *β* sheet region, whereas its negative side interacted with His13, Lys16, Val18 and Phe20. Due to its overall neutrality, Janus NP has almost zero Coulombic coupling with the *β* sheet, since the attractive and repulsive coupling energies tend to cancel each other (special orientation). Nevertheless, the vdW coupling energy (on average of −46 kcal/mol) keeps Janus NP attached on the *β* sheet surface. Finally, when the negatively charged NegNQ NP was positioned close to the *β* sheet, it gained a strong repulsive Coulombic coupling energy (of ≈200 kcal/mol) and a weak attractive vdW coupling energy (of −60 kcal/mol) (Fig. S3), due to the presence of the NQTrp molecules, which prevented NegNQ NP from nesting on the fibril surface.

In short, Pos NP was initially nested on several Glu22 or Asp23 residues, but later it moved away to similar residues in different chains. Initially, the nature of binding of PosNQ, Janus and Pep NPs was quite similar like for Pos NP. For Janus, two types of ligands were interacting with oppositely charged residues, which increased their contact area. PosNQ and Pep NPs slowly extended their ligands to increase the number of contacts after their initial nesting.

**Figure 4:**
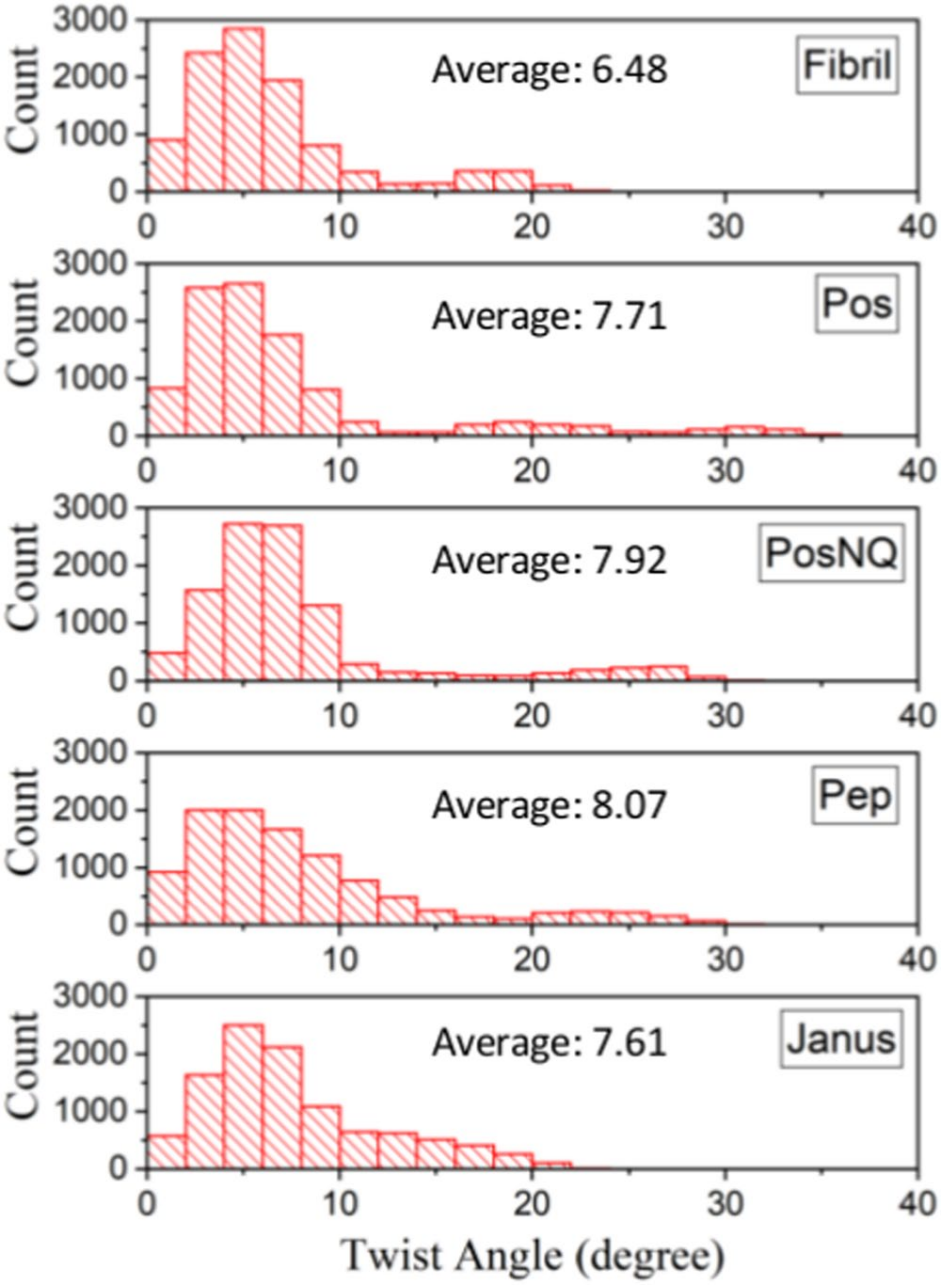
The distribution of the twist angles of the peptides in the absence (Fibril) and presence of each type of NP (Pos, PosNQ, Pep and Janus), calculated during the last 5 ns of trajectories.

In order to examine how the NP-coupling affected structural properties of the fibril, we calculated the average twist angle between two adjacent peptides in the absence/presence of NPs. In each peptide, we defined a vector going from the *α*-carbon atom of a 32^nd^ amino acid residue to the 18^th^ residue and measured the angle between two vectors of two neighboring peptides. 22 inner peptides out of 29 were used for calculation (see Methods). The simulation revealed that in the absence of NPs the average twist angle of the fibril is ≈6.48°. In the presence of NPs, the average twist angles of the interacting protofilament has increased to more than 7.5° (Pos: 7.7°, PosNQ: 7.93°, Pep: 8.08° and Janus: 7.62°) (Fig. 4). Figure 4 shows the distribution of twist angles in the last 5 ns, where the total number of counts in each plot is 10,500 (500 frames, each one contains 21 angles) and Fig. S5 shows average twist angles of each frame for all five systems. In all the cases, most of the twist angles are below 10° (fibril: 85.36%, Pos: 82.5%, PosNQ: 83.8%, Pep: 74.5%, Janus: 75.5%), but the presence of NPs extends the distribution tails. In Pos, PosNQ and Pep NPs, 9.3%, 8.7% and 8.6% of twist angles are ≥20°, respectively, whereas without NPs it is only 1.4%. The Janus NP has the largest percentage (23.33%) of twist angles of 10–20°. This enhancement of the twist angle was also observed when a D-enantiomeric amino acid peptide *D*3 (Arg-Pro-Arg-Thr-Arg-Leu-His-Thr-His-Arg-Asn-Arg) interacted with the *Aβ* fibril^25^.

We also evaluated the average fibril-NP contact area for NPs (Pos, PosNQ, Janus and Pep) with a favorable binding interaction to the fibril *β*-sheet to find out possible correlations with an induced twist of the protofilament. Figure S7 shows contact areas of NPs-fibril and NPs-fibril *β*-sheet (residue 12 to 40) over the last 5 ns. While the contact areas of Pos NP (full fibril: 6.83 *nm*^2^, fibril *β*-sheet: 4.43 *nm*^2^) are the smallest, the contact areas for NP-fibril *β*-sheet for PosNQ (fibril *β*-sheet: 13.59 *nm*^2^) and Janus (fibril *β*-sheet: 11.07 *nm*^2^) are comparable but 2.5–3 times larger than for Pos, due to the presence of two different types of ligands. Though the contact area of Pep NP with fibril *β*-sheet (fibril *β*-sheet: 10.01 *nm*^2^) is comparable to PosNQ and Janus, Pep NP contact area with full fibril (27.79 *nm*^2^) is the largest, as it has the longest ligands of all NPs studied, which make many contacts with the random coil regions of the fibril. The results show that NPs-fibril contact areas (Fig. S7) do not correlate with the induced twist angles in fibril *β*-sheet (Fig. 4). The twist angles induced by Pos, PosNQ, Janus and Pep are very similar, even though the Pos and Pep NP-fibril contact areas are significantly different than for the other two NPs.

We have also modeled coupling of NPs to the fibril tip, to find NPs that are potentially able to stop the fibril growth. From the five tested NPs, only NegNQ couple to the fibril tip, with an average Coulombic and vdW coupling energies of −108.6 and −35.1 kcal/mol, respectively (Fig. 3(A,B)). The positive (Pos, PosNQ, Pep) and Janus NPs experience a strong electrostatic repulsion at the tip (Fig. S4). Figure S6 shows selected equipotential surfaces of the *Aβ*40 fibril, where the light pink color (*β* sheet region) corresponds to −10.4 V and the light blue color (at the tip) corresponds to 2.6 V. This explains why positively charged NPs can be bound to the *β* sheet surface, whereas negatively charged NPs prefer to interact with the tip.

We used the same size of gold core (2.2 nm diameter) for all NPs, whereas the ligands have quite different lengths, especially in Pep NP. Free A*β* monomers can be easily adsorbed on the Pep NP ligands, due to their similar amino acid sequences, leading to significant vdW coupling. On the other hand, PosNQ and Pos NPs have a strong Coulombic coupling to free peptides (Fig. S2). Increasing their ligand length might increase the number of free peptides bound on PosNQ and Pos NPs at the cost of their reduced solubility, enhanced undesired protein interactions and smaller exposure of terminal charged groups. Addition of methylene groups (–*CH*_2_–) in the ligands can reduce their solubility and increase undesired protein interactions, whereas addition of ethylene glycol units can decrease the exposure of terminal $N{H}_{3}^{+}$ groups. The exposed surface area of ligated PosNQ and Pos can be comparable to Pep NP by increasing NPs core size, which have more ligands and larger total surface charges, possibly causing larger toxicity^26^ in biological systems.

From the estimated size of the NP-fibril contact area, it is clear that the induced twist angle of the fibril *β*-sheet is not proportional to the contact area, which shows that functional group-specific Coulombic couplings are the primary controlling factor for changing the twist angle. In this context, NPs with smaller cores could be more effective for biomedical applications. By reducing their core size, the overall curvature of NPs would increase, which could cause more stretching of some of the ligands and increasing of the force with which they can act on the fibril surface through multivalent coupling^10^. This stronger coupling might induce larger twists of the fibril closer to the NPs.

## Conclusion

In summary, the coupling of different NPs with the *Aβ*40 amyloid fibril was modeled by atomistic MD simulations. The results show that only Pep NP strongly couples with free peptides, and potentially affect their fibrillation. On the other hand, the positively charged NPs couple to the *β* sheet surface of the *Aβ*40 fibril, and locally deform it (helical twist), whereas the negatively charged NP couples to the tip of the fibril, and can potentially affect its growth. The inhibitor (NQTrp) molecules provide a number of heavy atoms coupling to amino acid residues, which increases the vdW coupling energy. The coupling of NPs to different polymorphs of the *Aβ* fibril might be different, due to different arrangements of exposed group sequences. These studies could help in designing nanomedicines for curing of neural diseases.

## Methods

The initial structure of an *Aβ*40 fibril was prepared based on a protein structure (pdb ID 2LMO)^27^. In the coordinate file, first eight amino acid residues of each peptide were missing, which were added as disordered chains by the Modeller program^28,29^. Then, the fibril was extended up to 13–14 nm (29 peptides in each protofilament). The prepared fibril was simulated in a 150 mM NaCl solution for 5 ns. NPs were modeled with icosahedral gold shell core of a 2.2 nm diameter, where 90 ligands were homogeneously distributed. Masses of gold atoms were rescaled to mimic a solid gold core. Zero partial charges were assigned to the gold atoms bonded together with rigid bonds (*b*_0_ = 2.74 Å and large *K*_*b*_). Ligands were attached to gold atoms via thiol groups. Angles, dihedrals in between ligands and gold cores were not considered. We placed the NPs at 5–10 Å above the fibril. The positively charged NPs were close to Glu22, Asp23 and the negatively charged NP was close to Lys16. In the case of Janus NP, the negative and positive surfaces were oriented towards Lys16/Glu22 and Asp23 respectively. Better selected initial positions of NPs reduce the exploration timescales for finding the NPs binding sites on the *β*-sheet surface of the fibril. All the simulated systems contained 400,000–600,000 atoms. After the initial minimization and warming to 300 K, ions and water molecules were equilibrated for 2 ns with restraining the movement of the NP core and the fibril backbone by a harmonic force constant of 1 kcal/mol Å^2^. Then, all the systems were simulated for 90–100 ns with releasing the NPs and the fibril. Visual Molecular Dynamics 1.9.3 (VMD) (https://www.ks.uiuc.edu/Research/vmd/)^30^ was used to visualize and create all the images of the ligands, NPs and the fibril.

The systems were simulated with a NAMD package^31^ using CHARMM general^32–34^ and protein forcefields^35–37^. In the simulations, we used a Langevin dynamics with a damping coefficient of *γ*_*lang*_ = 0.1 *ps*^−1^. Nonbonded vdW interactions were calculated with the Lennard-Jones (12,6) potential using a cut-off distance, *d* = 10 Å. Long range electrostatic interactions were calculated by the PME method^38^ in the presence of periodic boundary conditions and the MD integration time step was set to 2 fs. The simulations were performed using an NPT ensemble with *P* = 1 bar and *T* = 300 K. The simulations of five NPs at the tip of the fibril were also performed using similar conditions.

We performed the simulations of NPs binding on the fibril surface for 100 ns timescale, which is long enough for NPs to explore the fibril surface during the initial association process and bind to it if favourable. This timescale is too short to describe a full equilibration of the fibril without or with NPs bound to it. After simulating each system, we calculated the interaction energies (electrostatic and vdW interaction energy) of the NPs with fibril using the VMD energy analysis plugin considering the dielectric constant of water equals to 78.5. The average was performed over last 5 ns (500 frames). Despite this limited equilibration (100 ns of pre-equilibration), averaging of the data collected over the last 5 ns was specifically selected to describe the final state of the system reached in simulation to reveal the nature of binding of NPs to the fibril. We investigated the effect of NPs coupling to the *β* sheet surface of the fibril by calculating the average twist angle between two peptides. To calculate the twist angles, we first defined a vector from alpha carbon atoms of 32^nd^ amino acid residue to 18^th^ residue of each peptide and measured the angle between the two vectors of two consecutive peptides^39^. We calculated the average of the twist angles over the last 5 ns (500 frames) of the simulation trajectories. We calculated the twist angles from 4^th^ to 25^th^ monomer of each layer. In the calculations, the terminal peptides were not taken into account to remove the boundary effect of a fibril simulation in a water box. The calculation is performed using equation ${\theta }_{twist}=1/m{\sum }_{j=4}^{24}{\phi }_{j,j+1}$, where *m* is the number of peptide pairs, *j* and (*j* + 1) are the indices of two consecutive peptides and ${\phi }_{j,j+1}$ is a twist angle for a pair of peptides. Each distribution of twist angles was prepared using 10500 datapoints collected from 500 frames (each frame contains 21 twist angles).

We have separately calculated the contact area of NPs (Pos, PosNQ, Janus and Pep) with a full fibril and with a *β*-sheet of the fibril. To estimate the contact area, solvent accessible surface area (SASA) of NPs in the absence of fibril, in the presence of fibril and in the presence of fibril *β*-sheet were calculated using VMD SASA plugin. In this calculation, a radius of the solvent of 1.4 Å was considered. A sphere of the combined radius (solvent radius + vdWs radius of NP atom) was imaged surrounding each NP atom and 500 points were randomly distributed on the surface of the sphere. All the points were checked against the surface of all the neighboring atoms and the accessible number of points was multiplied with the assigned surface area that each point represents. In the case of NPs lacking the fibril, all the points at the sphere surface were checked against the surface of neighboring NP atoms, whereas in the case of NPs in the presence of fibril, the points were also checked against the fibril atom surface. Subtracting the SASA value of NPs in the presence of fibril and in the presence of fibril *β*-sheet from the NP SASA in the absence of fibril gave us the NP contact area with fibril and fibril *β*-sheet, respectively. In Fig. S7, we reported the average contact area of NPs with fibril and NPs with fibril *β*-sheet. The averaging was performed over last 5 ns (500 frames).

In the case of NP with free peptides, five *Aβ*40 peptides are randomly distributed surrounding each NP. The systems were kept in a 150 mM NaCl solution. At the beginning of the simulations, ions and water molecules were equilibrated for 2 ns. Then each system was simulated for 100 ns with the similar conditions like NP-fibril systems to identify the NPs that can capture freely solvated peptides due to their high mutual affinity. Then, we calculated the interaction energies of each peptide with NP surfaces using VMD energy analysis plugin considering water dielectric constant 78.5. We also calculated the number of heavy atoms of each peptide within 5 Å of the NP surfaces. All the analyses were performed on the last 5 ns (500 frames) of the simulations.

## Supplementary information

Supplementary information
