# Supplementary material for: Computational screening of nanoparticles coupling to Aβ40 peptides and fibrils
Source: Sci Rep. 2019 Nov 28;9:17804. doi: 10.1038/s41598-019-52594-8 (PMC6883061; doi:10.1038/s41598-019-52594-8)
Supplement: Supplementary file 1 — Supplementary information [file 41598_2019_52594_MOESM1_ESM.docx]

Supporting Information

Computational screening of nanoparticles coupling to Aβ40 peptides and fibrils

Soumyo Sen^1^, Lela Vuković^2^, and Petr Král^3^

^1^University of Illinois at Urbana-Champaign, NIH Center for Macromolecular Modeling and Bioinformatics, Beckman Institute, Urbana-Champaign, 61801, United States

^2^University of Texas at El Paso, Department of Chemistry and Biochemistry, El Paso, 79968, United States

^3^University of Illinois at Chicago, Departments of Chemistry, Physics, Biopharmaceutical Sciences and Chemical Engineering, Chicago, 60607, United States

pkral@uic.edu, lvukovic@utep.edu


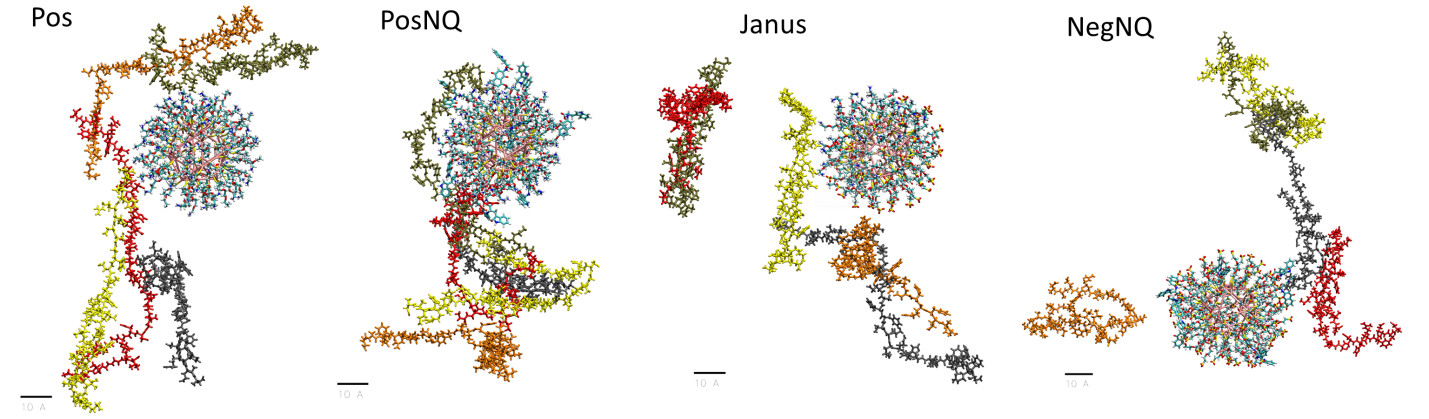
Fig S1: Free Peptides with NPs. Images of molecular structures created by VMD 1.9.3 software (http://www.ks.uiuc.edu/Research/vmd/):


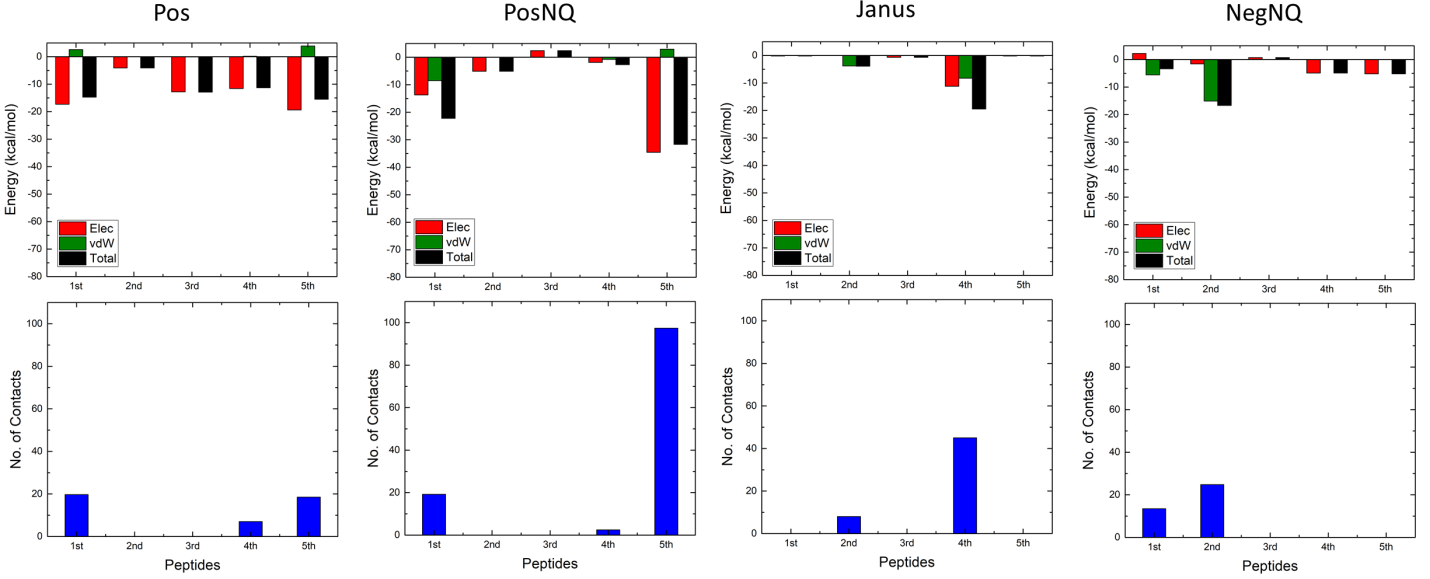
Fig S2: Interaction energies and number of heavy atom contacts of each free peptide with NPs:

Fig S3: Interaction energies of each NP with the βsheet surface of Aβ40 fibril:


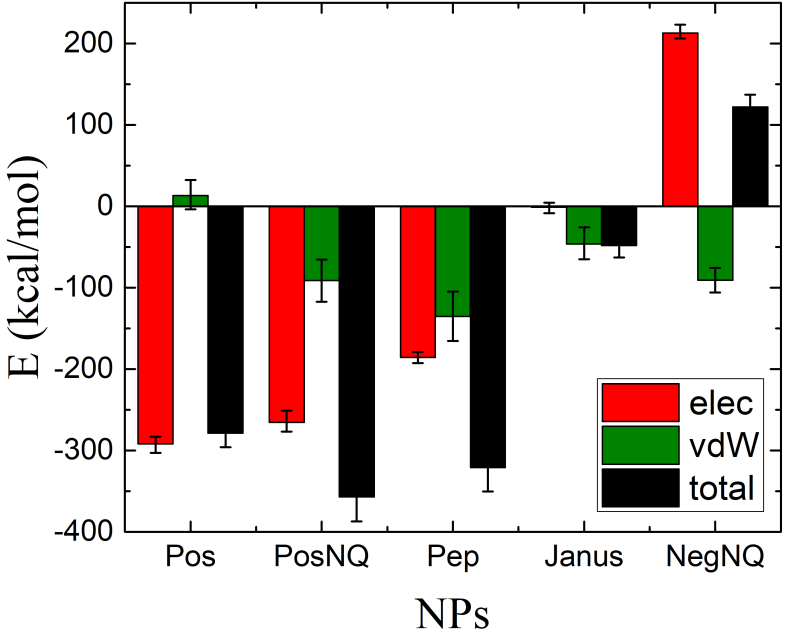


Fig S4: Interaction energies of each NP with the tip of Aβ40 fibril:


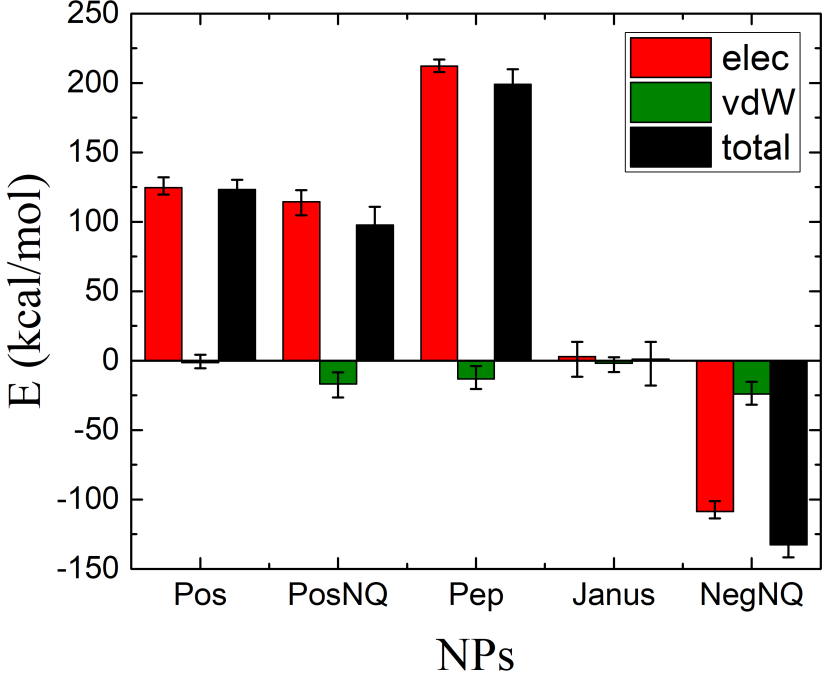


Fig S5: Average twist angles of the peptides (22 inner peptides) of the fibril protofilament interacting with NP:


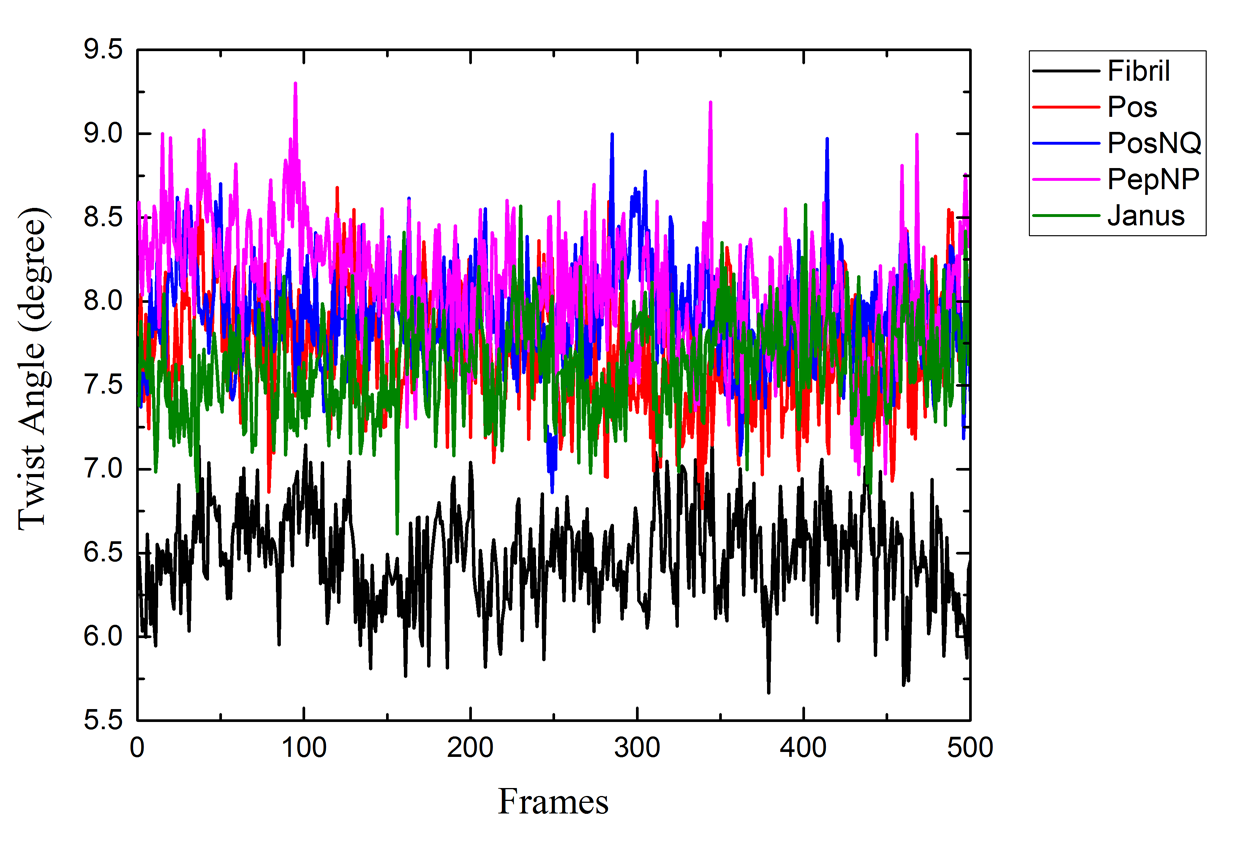


Fig S6: Electrostatic potential surfaces of Aβ40 fibril. Pink: -10.4 V and light blue: 2.6 V. Images of molecular structures and surfaces created by VMD 1.9.3 software (http://www.ks.uiuc.edu/Research/vmd/):


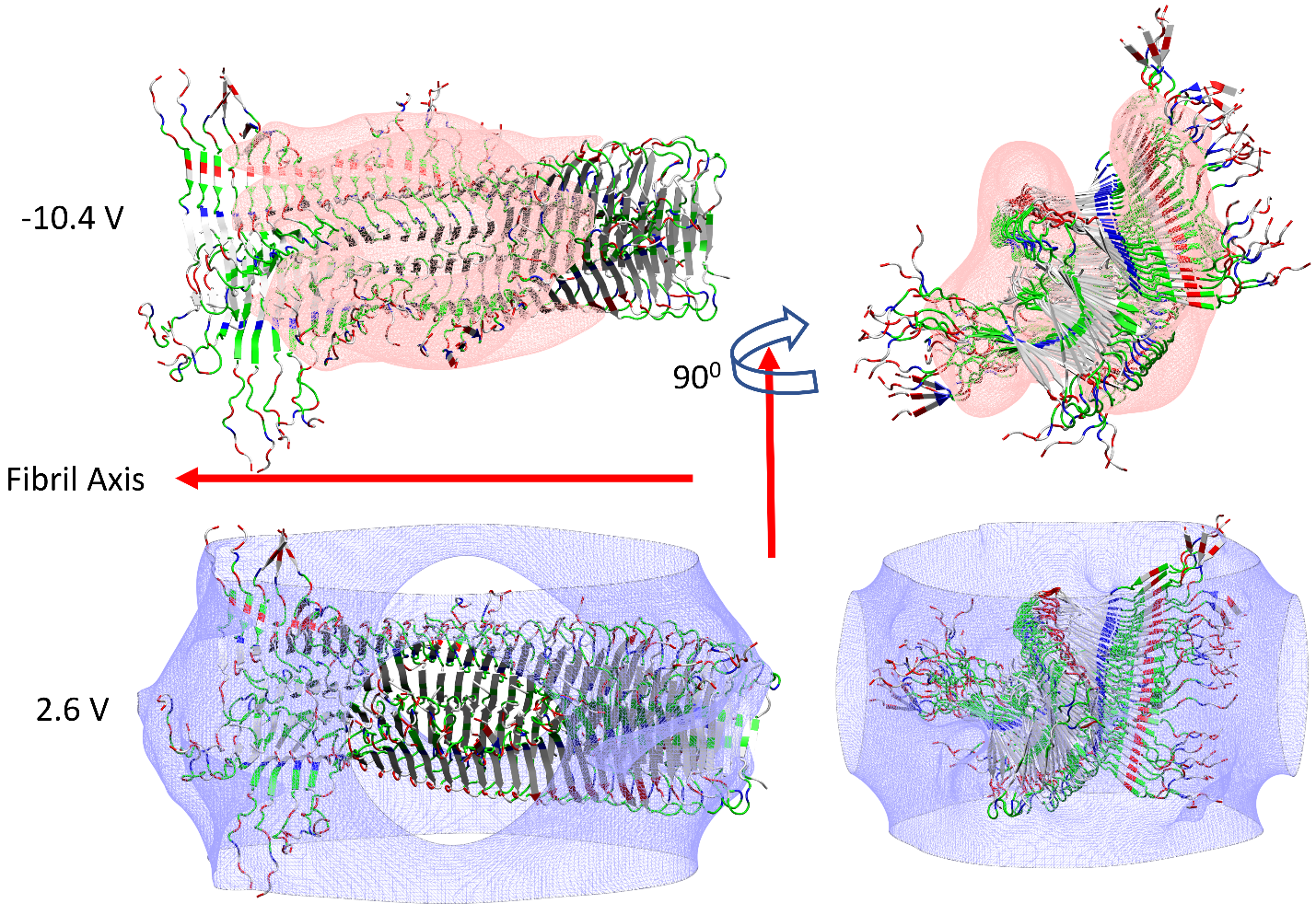


Fig S7: Contact areas of NPs with full fibril and fibril β-sheet separately

| NPs | Contact Area of NPs with Full Fibril (nm^2^) | Contact Area of NPs with β-sheet Portion (residue 12 to 40) of Fibril (nm^2^) |
| --- | --- | --- |
| Pos | 6.83 | 4.43 |
| PosNQ | 19.17 | 13.59 |
| Janus | 12.82 | 11.07 |
| Pep | 27.79 | 10.01 |
